# High-Pressure Insertion of Dense H_2_ into a Model Zeolite

**DOI:** 10.1021/acs.jpcc.1c02177

**Published:** 2021-03-29

**Authors:** Wan Xu, Xiao-Di Liu, Miriam Peña-Alvarez, Hua-Chao Jiang, Philip Dalladay-Simpson, Benoit Coasne, Julien Haines, Eugene Gregoryanz, Mario Santoro

**Affiliations:** † Key Laboratory of Materials Physics, Institute of Solid State Physics, HFIPS, Chinese Academy of Sciences, Hefei 230031, China; ‡ University of Science and Technology of China, Hefei 230026, China; § Centre for Science at Extreme Conditions & The School of Physics and Astronomy, The University of Edinburgh, Peter Guthrie Tait Road, Edinburgh EH9 3FD, U.K.; ∥ Center for High Pressure Science & Technology Advanced Research, 1690 Cailun Road, Shanghai 201203, China; ⊥ Université Grenoble Alpes, CNRS, LIPhy, Grenoble 38000, France; # ICGM, CNRS, Université de Montpellier, ENSCM, Montpellier 34095, France; ¶ Istituto Nazionale di Ottica (CNR-INO) and European Laboratory for Non Linear Spectroscopy (LENS), Via N. Carrara 1, Sesto Fiorentino 50019, Italy

## Abstract

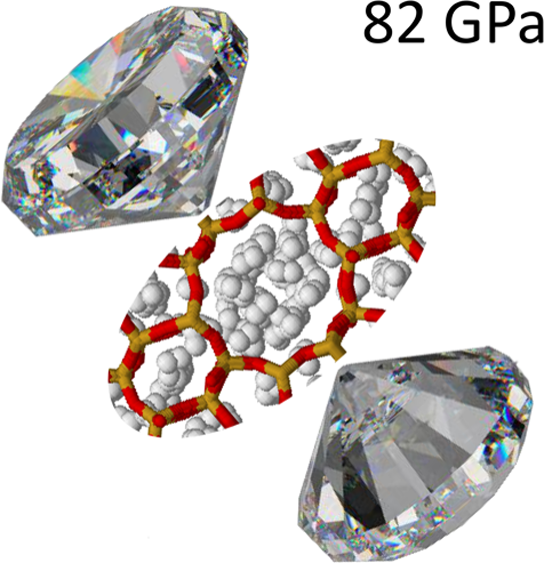

Our combined high-pressure synchrotron X-ray diffraction and Monte Carlo modeling studies show super-filling of the zeolite, and computational results suggest an occupancy by a maximum of nearly two inserted H_2_ molecules per framework unit, which is about twice that observed in gas hydrates. Super-filling prevents amorphization of the host material up to at least 60 GPa, which is a record pressure for zeolites and also for any group IV element being in full 4-fold coordination, except for carbon. We find that the inserted H_2_ forms an exotic topologically constrained glassy-like form, otherwise unattainable in pure hydrogen. Raman spectroscopy on confined H_2_ shows that the microporosity of the zeolite is retained over the entire investigated pressure range (up to 80 GPa) and that intermolecular interactions share common aspects with bulk hydrogen, while they are also affected by the zeolite framework.

## Introduction

Zeolites are archetypal microporous crystalline systems both natural and synthetic^1−4^ with a broad range of industrial applications. Adsorption studies on zeolites at extreme conditions led some of us and other groups to develop a rich and dynamic research area. Particularly, the high-pressure insertion of simple dense molecular systems as the guest species in zeolite hosts leads to the formation of exotic guest phases and unique properties for the host framework.^5−20^ Once the latter is supported by inserted guests to form a “molecular spring”, it resists the applied pressures. However, what would happen if the zeolites are filled by the most penetrating fundamental systems and also the most abundant element in the universe: hydrogen? Indeed, dense H_2_ is a “master” and benchmark system in high-pressure sciences.^21^ Dense, subnano confined states of H_2_ could be investigated and compared to those of bulk hydrogen at extreme conditions, since these states would add to the general view of such a fundamental element. The highly penetrating character of H_2_ could be also compared to other larger molecular and atomic systems to allow us to investigate the ultimate capability of filled zeolites to resist the pressure-induced pore collapse and the consequent pressure-induced amorphization (PIA). The complete deactivation of PIA can then provide information on the local structure of the framework cation, silicon in our case, at record extreme conditions. Indeed, it is of great interest to search for the extent to which the 4-fold coordination of silicon can be preserved. In bulk silica, the thermodynamic 4-fold to 6-fold transformation occurs below 10 GPa, while metastable 4-fold coordinated phases can survive at most up to 20–40 GPa.^22−25^ In gas hydrates, similar host–guest systems, the four-connected framework is built up by H-bonded water molecules, instead of being covalently bonded like in zeolites, and it is filled by simple gaseous molecules. Recently, CH_4_-filled ice has been found to be stable up to a record pressure of 150 GPa.^26^ On the other hand, cold compression of methane clathrates at GPa has been found to lead to PIA with the amorphous form still being a host–guest gas hydrate.^27^

**Figure 1:**
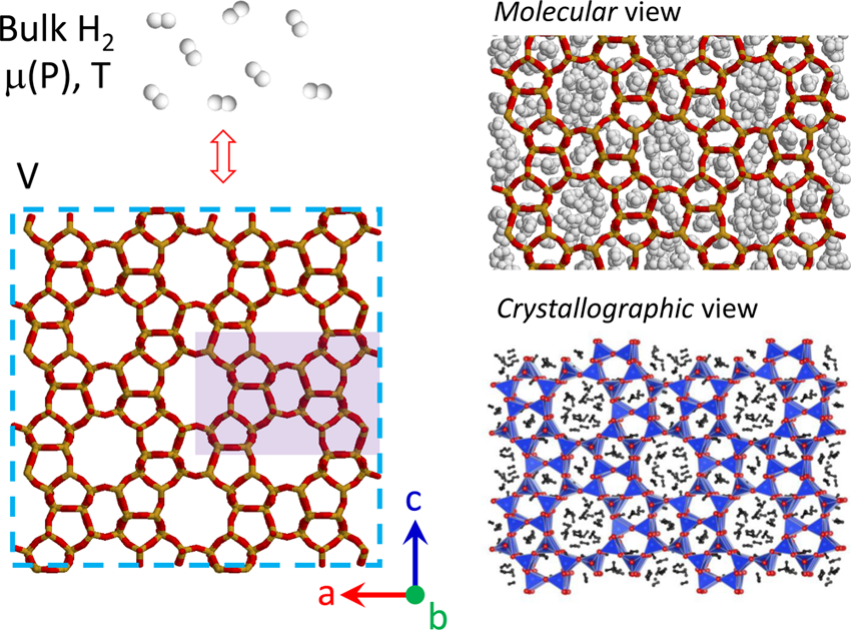
Structure of H_2_-filled orthorhombic silicalite-1. Left: principles of MC modeling in the grand canonical ensemble. Like in real experiments, a zeolite material is set in contact with an infinite reservoir of bulk H_2_. The purple shaded area denotes the unit cell volume, while the blue dashed line indicates the periodic boundary conditions. Right: molecular (top) and crystallographic (bottom) view of H_2_ inserted in silicalite-1 at room temperature and *P* = 7 GPa. In addition to the channels, some hydrogen molecules occupy the cages enclosed by the small 5- and 6- membered rings. In the molecular view, the red and orange sticks correspond to the chemical bonds between silicon and oxygen atoms, while the white spheres correspond to the hydrogen atoms in H_2_ (the sphere radius roughly corresponds to the van der Waal’s radius of hydrogen). In the crystallographic view, the blue tetrahedra correspond to the silica tetrahedra in silicalite-1 with the red spheres showing the apical oxygens. The black dumbbells represent the inserted H_2_ molecules. A 2 × 2 × 2 unit cell is considered here for the sake of clarity.

We focused our work on a model, pure SiO_2_ zeolite, silicalite-1 in order to avoid catalytic effects and to investigate how the pore size and shape affect the topology of the confined molecular form under pressure. Silicalite-1 is characterized by a framework of 4-, 5-, 6-, and 10-membered rings of corner-sharing SiO_4_ tetrahedra forming interconnected, mutually orthogonal straight and sinusoidal channels, with ambient pressure diameters of close to 5.5 Å (see Figure 1, at 7 GPa, together with inserted H_2_).^28,29^ Silicalite-1 is produced in crystals of several tens of microns, which makes it very suitable for optical spectroscopy studies.

In this work, we show the high-pressure insertion of H_2_ in silicalite-1 up to 82 GPa. Based on synchrotron X-ray diffraction (XRD) and Monte Carlo (MC) modeling, we show that the insertion leads to an exotic glassy-like form of molecular hydrogen. H_2_-filled silicalite-1 is found to be stable up to at least 60 GPa and significantly less compressible than the same zeolite filled by bigger molecules; this is a consequence of the more penetrating nature of H_2_. The number of inserted molecules versus pressure is non-monotonic with a maximum close to two per SiO_2_ unit. Raman spectroscopy shows that the framework porosity is retained up to at least 82 GPa, and it provides important clues on intermolecular interactions.

Our experimental and theoretical methods are described in the Supporting Information(30) together with the relevant refs.^20,31−50^

## Results and Discussion

**Figure 2:**
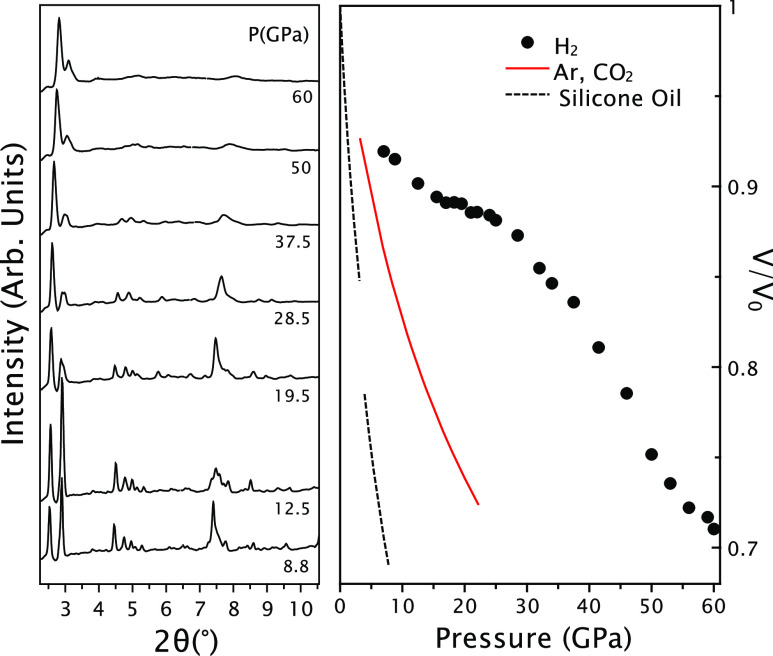
Left panel: selected powder XRD (λ = 0.4828 Å) patterns of H_2_-filled silicalite-1, measured upon increasing pressure. A broad Compton scattering background due to air and diamonds has been subtracted. Right panel: experimental pressure behavior for the relative to ambient pressure unit cell volume of H_2_-filled silicalite-1 (filled circles). Error bars are within the size of the circles. Red line: compression curve for Ar and CO_2_-filled silicalite-1.^8^ Black line: equation of state for silicalite-1, measured in a nonpenetrating pressure-transmitting medium silicone oil, where amorphization has been found to occur below 10 GPa.

Powder XRD patterns of H_2_-filled silicalite-1 were measured upon increasing pressure up to 60 GPa (Figure 2), in order to investigate to which extent the framework is stable at high pressures and also to provide inputs for MC modeling aimed to determine the amount of stored hydrogen. The large, nanometer scale, unit cell size of silicalite-1 is related to the microporosity of the framework, and it gives rise to the 101, 011, 200, 020, and 111 diffraction peaks located at very low 2θ angles, down to 2°–3°. The widths of the Bragg peaks increased gradually by a factor of 2 over the entire pressure range investigated. This can be linked to a gradual increase in deviatoric stress in the solid H_2_ pressure medium. We also observed some apparent changes in relative intensity principally due to the separation of overlapping peaks resulting from changes in the unit cell parameters (Figure S3 and Table S1). Importantly, at the highest pressures, in spite of the increase in line width, the peaks retained much of their initial integrated intensity (typically 30–100%) at low pressure. That suggests that silicalite-1 remains crystalline up to the highest pressure and, consequently, full 4-fold coordination of silicon by oxygen was retained at record pressures among all known group IV compounds, except carbon. Indeed, the thermodynamic transformation pressure from 4-fold to 6-fold coordination in bulk silica is lower by 1 order of magnitude. LeBail fitting of the XRD patterns (Figure S4) was used to obtain the pressure behavior of the unit cell volume of H_2_-filled silicalite-1 (Figure 2) normalized to its ambient pressure value. We can compare this compression curve with those observed for other penetrating simple gaseous systems such as Ar and CO_2_.^8^ The most striking result is the much higher relative volumes of up to 24% at 25 GPa measured for H_2_-filled silicalite-1 than for Ar or CO_2_ filled silicalite-1, clearly indicating that H_2_ is much more penetrating than the other two larger systems. Also, the compression curve of H_2_-filled silicalite-1 shows an anomaly. Indeed, a quasi-horizontal inflection point appears at around 19 GPa. Above this point, the curve is convex rather than concave, up to about 50 GPa. This anomaly is suggestive of substantial pressure changes in the filling of silicalite-1 by H_2_, a hypothesis that can only be tested on the grand canonical ensemble-based MC model, whose main outputs are the spatial distribution and the number of inserted molecules per unit cell.

**Figure 3:**
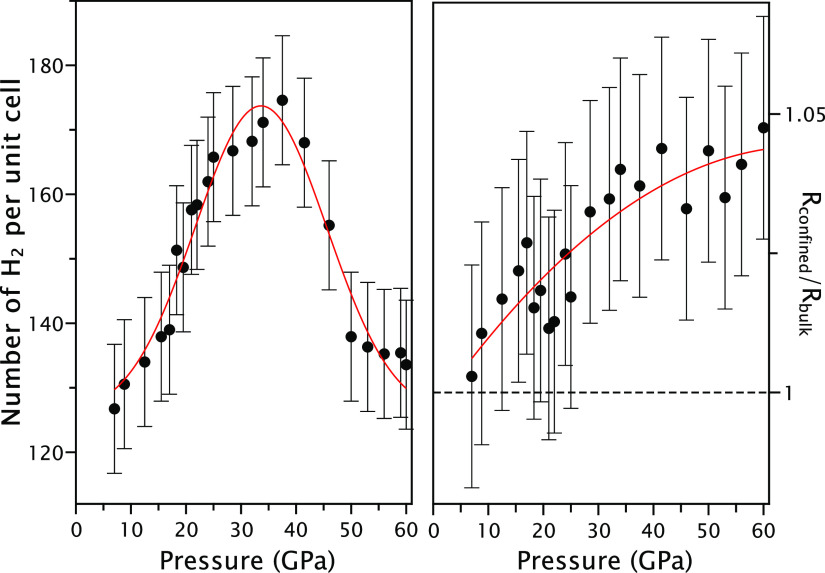
MC calculated pressure behavior of the number of inserted H_2_ molecules in silicalite-1 (left) and of the ratio of the nearest neighbor’s H_2_–H_2_ distance for confined hydrogen to that of bulk hydrogen (right). Error bars are due to the simulation and to the evaluation method of the average distances. Red lines through the points are guides for the eye.

In Figure 1, we report the structure of H_2_-filled orthorhombic silicalite-1 at 7.0 GPa, obtained by combining the experimentally determined lattice parameters with MC modeling. The zeolite framework is entirely filled by guest H_2_ molecules, 127 per unit cell. The state of inserted hydrogen is remarkable. Indeed, this is a disordered, dense form within the whole investigated pressure range by XRD and MC, that is up to 60 GPa. Also, considering the very large density reached in nanoconfined H_2_ at such extreme pressures, we assume this disordered form to be glassy-like rather than liquid-like. This type of disorder is supported by the intermolecular radial distribution function (RDF), reported in Figure S2 for a selected pressure of 7.0 GPa. As expected for glassy-like, that is disordered configurations, the RDF displays a strong peak at short distance (here about 2.6 Å) corresponding to the nearest neighbors’, followed by additional much weaker and broader peaks at increasing distances. Overall, the different peaks are broad with an amplitude that strongly decreases with increasing the intermolecular distance. The calculated number of inserted H_2_ molecules per unit cell of silicalite-1 by MC as a function of pressure clearly exhibits a maximum (Figure 3, left panel), which corresponds to the anomaly experimentally observed for the pressure behavior of the unit cell volume. The maximum is equal to about 174, and it is located at around 34 GPa. The very low compressibility of H_2_-filled silicalite-1 in the 15–25 GPa pressure interval is now found to be due to H_2_ rapidly entering silicalite-1 to a greater and greater extent upon increasing pressure in this range; here, the filling of H_2_ hinders the reduction of the host framework volume by strongly reducing the compressibility of the pores. Then, above 30–40 GPa, some amount of previously inserted H_2_ is extruded and the volume of silicalite-1 decreases as a result of the combined effect of guest extrusion and normal compression of the filled framework material. H_2_-filled silicalite-1 is thus a hydrogen-rich material, with H_2_ being physisorbed by the zeolite and an overall pressure-dependent chemical composition SiO_2_(H_2_)_*x*_, with *x* = 1.32–1.82. In fact, this gas content is substantially greater, in terms of the number of stored guest molecules, than that experimentally observed so far in any gas hydrate phase under any pressure-temperature condition,^51,52^ as may be expected as zeolites contain both cages and channels.

At least two remarkable differences emerge between H_2_-filled silicalite-1 and the filling of this zeolite with larger simple systems such as Ar and CO_2_.^53^ First, the maximum number of confined H_2_ molecules exceeds that for bigger molecules by a factor of 3–4. Second, H_2_ is so much more penetrating that even the cages with small openings of 1.4–3.0 Å diameter built up by 5- and 6- membered rings are well filled in this case. We found that the number of H_2_ molecules in these cages is equal to 20 per unit cell over the whole investigated pressure range, which is 11–16% of the total amount of inserted molecules. Also, we found that multiple filling of some cages occurs, similarly to several gas hydrates.^51,52,54,55^ Interestingly, the non-monotonicity for the pressure behavior of the filling appears to be entirely due to molecules inserted in the channels. All this explains why the volume of the zeolite is so much larger when H_2_, rather than bigger guests, is inserted into the framework.

Importantly, the nearest neighbor’s intermolecular distance for confined hydrogen is larger than that for bulk hydrogen (Figure 3, right panel) by a few percent, indicating that the H_2_-silicalite-1 interaction adds a negative term to the local pressure.

**Figure 4:**
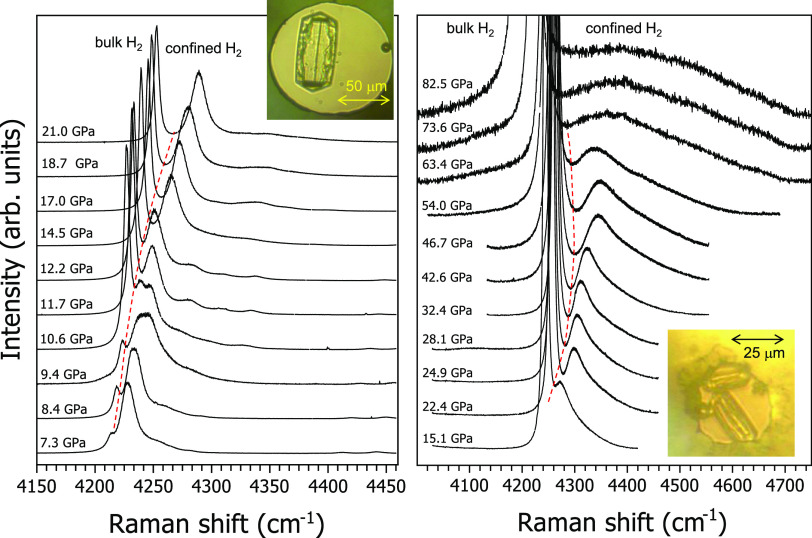
Selected Raman spectra of H_2_-filled silicalite-1 crystals in the frequency range of the H_2_ vibron, measured upon increasing pressure. Spectra measured on top of a silicalite-1 crystal of initial size (left) 80 × 40 × 40 μm^3^ and (right) 30 × 15 × 15 μm^3^. Red dashed lines separate the bulk and the confined H_2_ peaks. Insets: silicalite-1 crystals in the gasket holes at (left) 0.4 and (right) 4.2 GPa immersed in H_2_ as the pressure transmitting medium. Ruby chips are also present for pressure measurements.

The Raman spectroscopy investigation provides direct information on dynamical properties of the confined dense form of hydrogen. In Figure 4, we report waterfalls of selected Raman spectra of the H_2_ vibron for H_2_/silicalite-1 samples, measured upon increasing pressure on silicalite-1 crystals up to 82.5 GPa. In the spectra, we observe the pure H_2_ peak due to bulk hydrogen layers surrounding the crystals and several blue-shifted extra peaks which can be easily attributed to confined dense H_2_. The blueshift, which increases with pressure, is the net result of the modified intermolecular interactions in confined hydrogen and the interaction between the guest molecules and the internal walls of the porous host. Spectra measured on larger silicalite-1 crystals show up to 4–5 partially resolved peaks for confined hydrogen (Figure 4, left panel), likely to be ascribed to H_2_ guest molecules located on distinct host crystallographic sites of silicalite-1 and, as a consequence, experiencing different interactions. These peaks are much broader than the peak of bulk H_2_, and they broaden upon increasing pressure till they merge. This finding is compatible with confined hydrogen being highly disordered around the different crystallographic sites corresponding to the distinct peaks, in full agreement with the combined XRD/MC outcome where confined H_2_ is indeed found to be in a glassy-like state.

**Figure 5:**
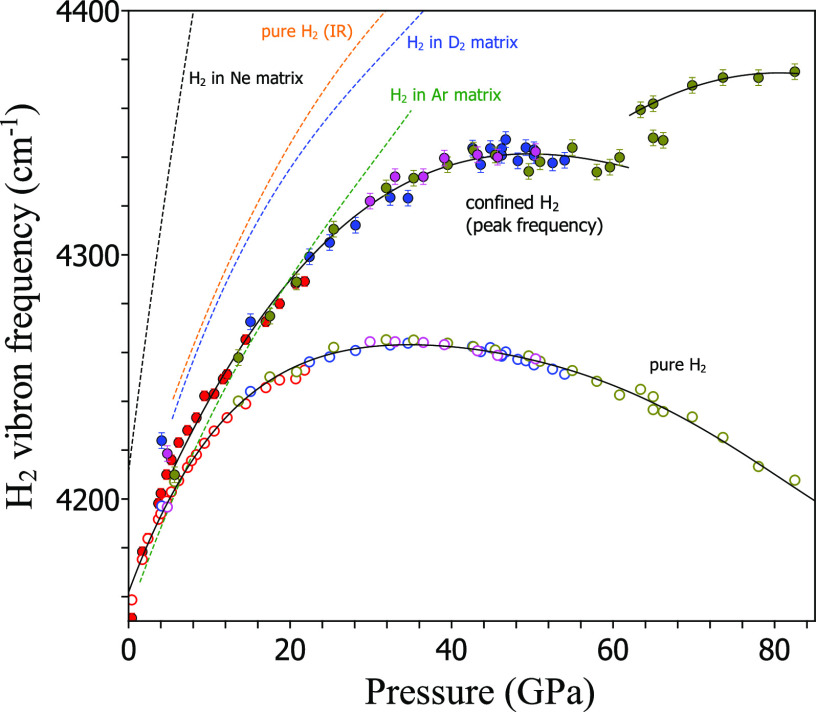
H_2_ frequency vs pressure for different materials and compounds. For this work, values for confined in silicalite-1 (full dots) and bulk H_2_ (open dots) (see text for details). Black lines: guides for the eye through these two data sets. Error bars are mainly due to the fitting procedure of peaks for confined H_2_. Other lines: pressure shift for H_2_ as a single molecule impurity in Ne (black), D_2_ (blue), and Ar (green) matrices, respectively.^56^ Orange line: pressure shift for the IR active H_2_ frequency in pure hydrogen.^57−59^

In Figure 5, we report the pressure shift of the H_2_ frequency for the most intense peak in confined H_2_ and for bulk H_2_ measured in this work up to 82.5 GPa and also for isolated H_2_ impurities in three crystalline matrices: Ne, Ar, and D_2_.^56^ The pressure behavior for the confined H_2_ frequency is remarkable; it is systematically higher than that of the Raman frequency for pure H_2_ and, more importantly, in both cases we observe a maximum, which is located at around 50 GPa in confined H_2_ and 35 GPa in pure H_2_. In addition, in confined hydrogen, a frequency jump occurs around 62 GPa, beyond the pressure range of our XRD investigation, which probably relates to a major structural change in silicalite-1, such as a yet unknown phase transition. The origin of the maximum in the H_2_ frequency for confined hydrogen can be easily traced back to the H_2_–H_2_ vibrational coupling^56−59^ (and references therein. See also SI). The Raman frequency being blue-shifted in confined H_2_ and the maximum being at higher pressures with respect to bulk H_2_ suggest that vibrational coupling is weaker in the confined form. This is due to the larger intermolecular distance (see Figure 3) and to the reduced number of H_2_ neighbors. An extreme case in this respect is that of molecules in the cages of silicalite-1, where the number of H_2_ neighbors drops to 1 or 0 and the vibrational coupling is nearly or entirely switched off. This case likely corresponds to the highest frequency components in the Raman frequency distribution for confined hydrogen (Figure 4). The Raman spectra of the rotational peaks (Figure S5) show that confined H_2_ is a near free rotor, the rotations of which are somewhat more hindered than for pure hydrogen.^60^

## Conclusions

Our investigation uncovered an exotic form of dense hydrogen inserted in a zeolite at pressures up to 80 GPa. What is the Mbar/multi-Mbar fate of H_2_-filled zeolites? What is the fate of silicon coordination in gas-filled framework materials and, more generally, of host–guest systems where the host is a covalent or H-bonded network and the guest is a simple atom or molecule, and what is the potential to synthesize novel H-rich systems in this way? All these questions among others build up an entirely unexplored field, initiated here and left to future intriguing studies.

## Supporting Information Available

Experimental and computational methods; supplementary GCMC results; supplementary XRD results; H_2_–H_2_ vibrational coupling; and supplementary Raman spectroscopy results (PDF)
