# Supplementary material for: High-Pressure Insertion of Dense H2 into a Model Zeolite
Source: J Phys Chem C Nanomater Interfaces. 2021 Mar 29;125(13):7511–7. doi: 10.1021/acs.jpcc.1c02177 (PMC9490752; doi:10.1021/acs.jpcc.1c02177)
Supplement: Supplementary file 1 — jp1c02177_si_001.pdf [file jp1c02177_si_001.pdf]

Supporting Information for: "High Pressure Insertion of Dense H_2 into a Model Zeolite"

Wan Xu,[†,‡] Xiao-Di Liu,[*,†] Miriam Peña-Alvarez,[¶] Hua-Chao Jiang,[†] Philip Dalladay-Simpson,[§] Benoit Coasne,[‖] Julien Haines,[⊥] Eugene Gregoryanz,[§,¶,†] and Mario Santoro[*,#,†]

†*Key Laboratory of Materials Physics, Institute of Solid State Physics, HFIPS, Chinese Academy of Sciences, Hefei 230031, China*

‡*University of Science and Technology of China, Hefei, China*

¶*Centre for Science at Extreme Conditions & The School of Physics and Astronomy, The University of Edinburgh, Peter Guthrie Tait Road, Edinburgh, U.K.*

§*Center for High Pressure Science & Technology Advanced Research, 1690 Cailun Road, Shanghai, 201203, China*

‖*Université Grenoble Alpes, CNRS, LIPhy, Grenoble, France*

⊥*ICGM, CNRS, Université de Montpellier, ENSCM, Montpellier, France*

#*Istituto Nazionale di Ottica (CNR-INO) and European Laboratory for non Linear Spectroscopy (LENS), via N. Carrara 1, 50019 Sesto Fiorentino, Italy*

E-mail: xiaodi@issp.ac.cn; santoro@lens.unifi.it

Experimental and computational methods

High purity dense H_2 was gas loaded in diamond anvil cells (DACs) at 200 MPa together with hydrophobic hydrogen-free silicalite-1 single crystals (SOMEZ, France)[1] or powders.[2] For Raman spectroscopy, we loaded 1-2 silicalite-1 crystals of several tens of micron with their long axis corresponding to the crystallographic c direction parallel to the diamond culet surface; instead, silicalite-1 powder was loaded for the XRD studies. In these samples, hydrogen was in two forms: confined H_2 in silicalite-1 and bulk H_2. DACs were equipped with 200-300 μm culet and ultralow fluorescence Ia diamonds and we used Re as the gasket material. The gasket hole had initial diameter and thickness of about 100 and 50 μm, respectively. Pressure was measured by the ruby fluorescence technique,[3] and also by the pressure shift of the Raman diamond edge[4] and of the H_2 vibron.[5]

Raman spectra on H_2-silicalite-1 mixtures were measured using the 532 nm line of a frequency doubled Nd:YAG laser as the excitation source. Backscattering geometry was used with a 20× micro-objective, with a few micron laser spot. The signal, once filtered by notch filters, was detected by a single Acton/SpectraPro 2500i monochromator, equipped with a CCD detector (Princeton Instruments, PIXIS: 400). The Raman spectral resolution was about 1 cm^{-1}. Raman spectra were measured on different points within the sample region for each pressure, both in the free H_2 bulk areas and on top of the silicalite-1 crystals, with an integration time of 300-600 s per point. Synchrotron powder XRD measurements on H_2-silicalite-1 mixtures were performed on the extreme conditions beamline P02.2 at the Petra III synchrotron (proposal ID: I-20181128, I-20190519 EC) using monochromatic X-ray beam with λ=0.4828 Å and a micron sized spot, and the scattered X-rays were detected by a Perkin Elmer XRD1621 (2048× 2048 pixels, 200 μm× 200 μm) detector. The diffraction patterns were analyzed and integrated using Dioptas.[6] LeBail fits of the one dimensional diffraction patterns were performed with the program Fullprof[7] in order to get the unit cell parameters of silicalite-1. Pressures were measured using the equation of state of gold.[8]

We also performed Grand Canonical Monte Carlo (GCMC) simulations of H_2 insertion

at T = 300 K in pure siliceous zeolite (silicalite-1, MFI). GCMC is a simulation method based on statistical mechanics in which a system having a constant volume V (the pore with the adsorbed phase) is set in equilibrium with an infinite bulk reservoir of particles imposing its chemical potential μ and temperature T. Following previous works,[9,10] the relationship between pressure P and chemical potential μ for bulk H_2 at 300 K was determined as follows. We performed GCMC simulations of bulk H_2 to obtain the relationship between chemical potential μ and density, $\rho(\mu)$. In parallel, the relationship $\rho(P)$ between pressure P and density ρ is determined using molecular dynamics simulations in the isobaric-isothermal ensemble (NPT), and we also found that it agrees well with the available experimental $\rho(P)$ as shown in figure SM1.[11–13] From $\rho(\mu)$ and $\rho(P)$, we can determine in a straightforward way $\mu(P)$. Then, several force fields are available in the literature to model the H_2 molecule including its adsorption on solid surfaces. In particular, quantum mechanics-based force fields including quantum effects to distinguish H_2/D_2 adsorption are available using the FeynmanHibbs effective potential formalism.[14–18] While such effective potentials are needed to include strong quantum effects at low temperature, other simple force fields are available when room temperature or above is considered. Such classical interaction potentials range from very simple (single Lennard-Jones potential) to more complex force fields (including several interaction sites with partial charges). In the present work, we selected the force field by Darkrim and Levesque,[19] which correctly describes the density vs. pressure curve at ambient temperature for pressures up to a few GPa, as shown here in figure S1. In this force field, the H_2 molecule is described as a rigid, diatomic molecule with the two H atoms separated by a distance 0.074 nm. A Lennard-Jones site is added at the molecule center of the molecule with the following interaction parameters: $\sigma = 0.2958$ nm, $\varepsilon/\kappa_B = 36.7$ K. To describe the quadrupole of the H_2 molecule, each H atom carries a charge $q= +0.468$ while the Lennard-Jones site carries a negative charge equal to -2q. Molecular interactions between the H_2 molecule and the O atoms of the MFI zeolite are also described using the Lennard – Jones potential (those with the Si atoms are neglected as the Si bears a much smaller polarizability than that of

the O atom). The cross parameters σ and ε were determined by combining the like-atom parameters using the Lorentz – Berthelot rules (the like-atom parameters for zeolite MFI are $\sigma = 0.300$ nm, and $\varepsilon/\kappa_B = 93.53$ K for O[20]). Following previous works on H_2 adsorption,[21] electrostatic interactions with the host zeolite were neglected as it were shown to be small compared to the dispersive energy corresponding to the attractive part of the Lennard-Jones interaction. The pressures (and therefore chemical potentials) considered in our simulation approach were set equal to those investigated in the experiments; at each pressure, the unit cell parameters determined experimentally were used to build the simulation box corresponding to zeolite MFI (2×2×3 unit cells were considered). The host structure was kept rigid and periodic boundary conditions were used along the x, y, z directions to avoid finite size effects.

SUPPLEMENTARY GCMC RESULTS

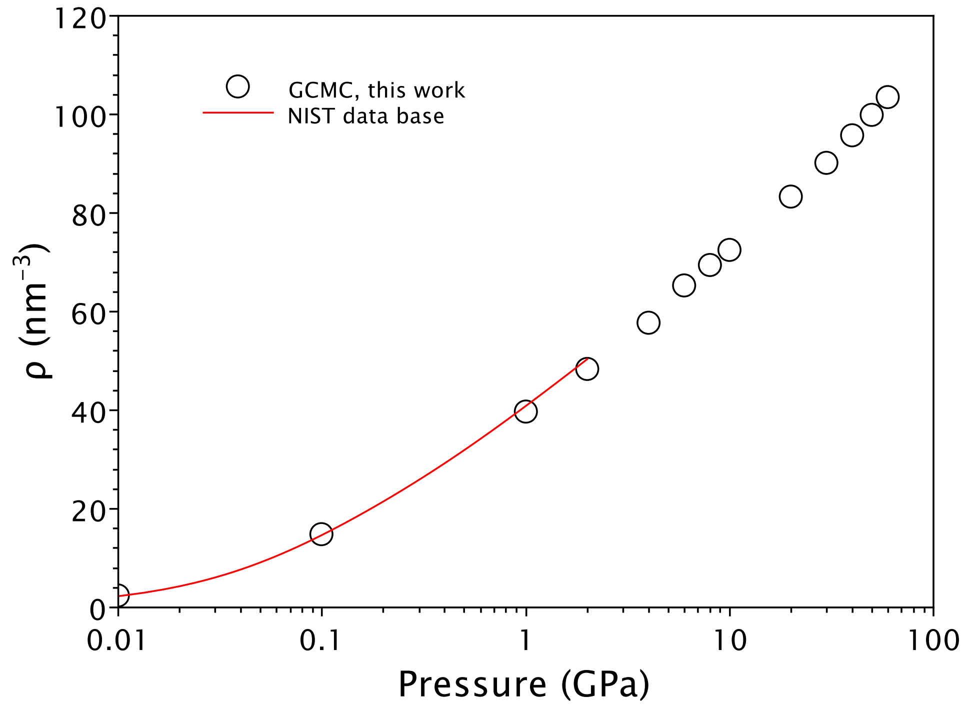

Figure S1: Pressure behavior for the numerical density, at 300 K, of pure molecular hydrogen calculated in this work by GCMC (empty circles) and from the NIST database[11–13](red line), respectively.

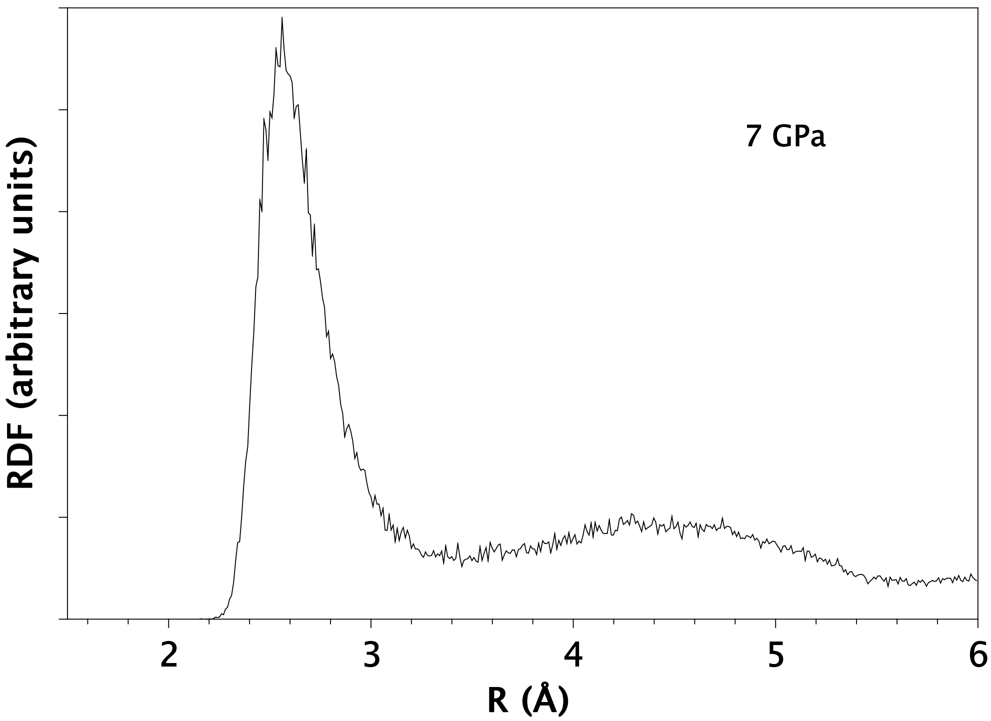

Figure S2: Radial distribution function for dense H_2 confined in the channels of silicalite-1 for a selected pressure of 7.0 GPa. The vertical axis has not been normalized.

SUPPLEMENTARY XRD RESULTS

Table S1: Experimentally obtained unit cell parameters of orthorhombic silicalite-1 (space group *Pnma*) in H_2 as a function of pressure. Note that the empty starting material is monoclinic with a=20.1344(1) Å, b=19.9018(1) Å, c=13.38641(8) Å, α=90.6207(5) ° in the non-standard setting of the space group $P2_1/n(P2_1/n11)$ in order to retain the same axes as the orthorhombic aristotype *Pnma*.[22]

P(GPa)	a(Å)	b(Å)	c(Å)	V(Å^3)
7.0	19.50(2)	19.28(4)	13.09(2)	4921(14)
8.8	19.49(1)	19.20(1)	13.09(1)	4898(4)
12.5	19.51(2)	19.09(1)	12.95(1)	4825(6)
15.5	19.53(2)	19.06(1)	12.86(1)	4786(7)
17.0	19.48(1)	19.03(1)	12.86(1)	4769(5)
18.3	19.32(1)	19.05(1)	12.96(1)	4770(5)
19.5	19.30(1)	19.06(1)	12.96(1)	4766(5)
21	19.26(1)	19.06(1)	12.91(1)	4740(5)
22	19.26(1)	19.05(1)	12.92(1)	4741(5)
24	19.28(2)	19.03(1)	12.9(1)	4732(6)
25	19.32(2)	19.03(1)	12.83(1)	4717(6)
28.5	19.33(1)	18.95(2)	12.75(1)	4672(7)
32	19.21(3)	18.88(2)	12.62(1)	4575(10)
34	19.19(2)	18.81(4)	12.55(2)	4530(12)
37.5	19.23(1)	18.79(3)	12.38(2)	4474(9)
41.5	19.10(4)	18.59(5)	12.22(2)	4340(17)
46	18.98(5)	18.28(1)	12.11(1)	4204(14)
50	18.49(5)	18.13(4)	12.00(2)	4023(16)
53	18.43(5)	17.88(8)	11.94(4)	3937(22)
56	18.37(7)	17.77(7)	11.84(2)	3865(22)
59	18.39(5)	17.74(4)	11.76(2)	3837(15)
60	18.27(6)	17.72(6)	11.74(2)	3802(20)

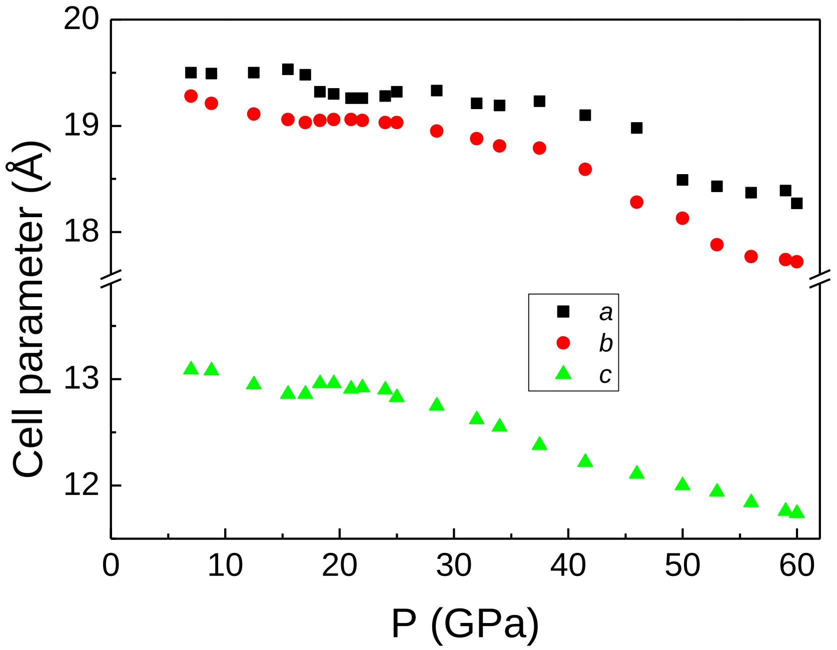

Figure S3: Experimental pressure behavior for the unit cell parameters of H_2-filled silicalite-1.

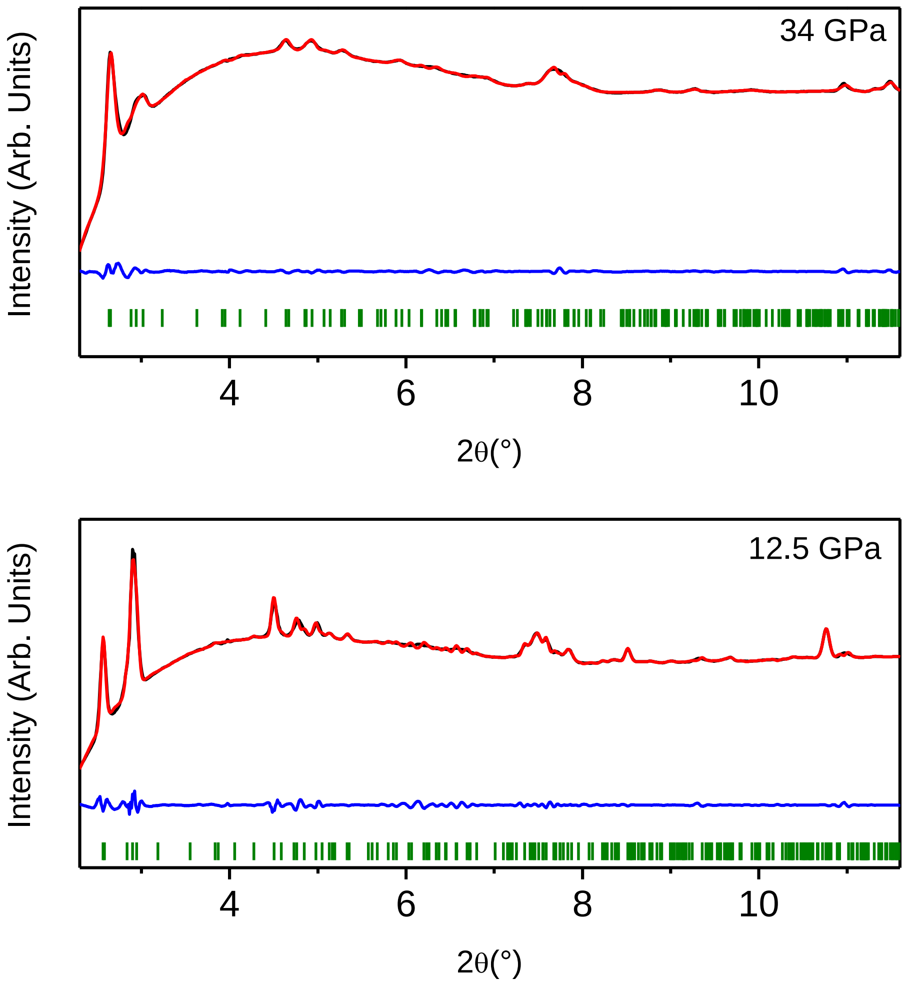

Figure S4: Selected LeBail fits to XRD (λ=0.4828 Å) patterns of H_2-filled silicalite-1. The sharp Bragg peaks of silicalite-1 are superimposed on the broad Compton scattering background due to air and diamonds. Experimental (black), calculated (red) and difference (blue) profiles for the Pnma unit cell of silicalite-1-H_2 at 12.5 GPa (below) and 34 GPa (above). Vertical bars indicate the calculated positions of the Bragg reflections.

H_2-H_2 VIBRATIONAL COUPLING

The origin of the maximum in the pressure behavior of the H_2 Raman frequency can be easily understood based on literature on pure hydrogen and H_2 impurities in matrixes[23–26] (and references therein). In isolated H_2 impurities in matrices, the frequency monotonically hardens with pressure as a trivial result of the density increase. Instead, in pure hcp solid

hydrogen, where two molecules are contained in the primitive cell, the intermolecular vibrational coupling interaction splits the H_2 frequency in two distinct crystalline modes: in-phase (Raman) stretching and out-of-phase (IR) stretching, respectively. The IR frequency is not directly affected by the vibrational coupling potential and it monotonically increases with pressure. Instead, the Raman frequency is affected by a subtractive vibrational coupling term, which makes it lower than the IR frequency. Also, the absolute strength of vibrational coupling rapidly increases with pressure until it bends down the pressure shift of the Raman frequency, hence the origin of the maximum described above.

SUPPLEMENTARY RAMAN SPECTROSCOPY RESULTS

In figure S5 we report selected, low frequency Raman spectra of H_2-filled silicalite-1 (black) and of bulk H_2 (red), measured upon increasing pressure. Spectra for pure H_2 are dominated by the four rotational lines S_0(J), with J=0, 1, 2 and 3. The spectra of H_2-filled silicalite-1 have been measured on top of a zeolite crystal, see figure 4 (left panel) in the main paper. In these spectra, we observe the overlapped rotational peaks of both confined and bulk H_2, where these last ones are due to pure hydrogen layers surrounding the crystal, along with the phonon peaks of the silicalite-1 framework. Rotational peaks for confined H_2 are much broader than those of pure H_2 at GPa, whereas the two sets of peaks rapidly broaden with pressure, those for pure H_2 more rapidly, in such a way that it is increasingly difficult to distinguish between them above 20 GPa.

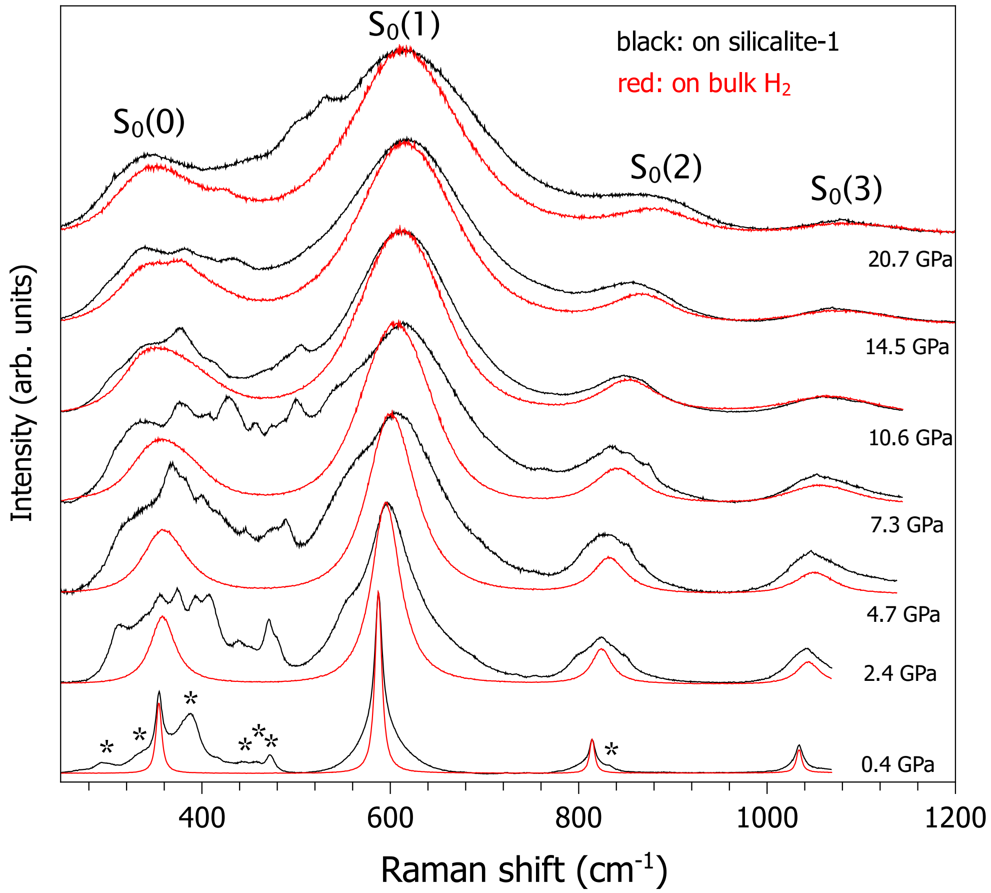

Figure S5: Selected, low frequency Raman spectra of H_2 filled silicalite-1 (black), and of bulk H_2 (red), measured upon increasing pressure. The $S_0(J)$ rotational peaks of H_2 are labelled. Phonon peaks of the silicalite-1 framework are marked by stars only at 0.4 GPa.

References

(1) Haines, J.; Levelut, C.; Isambert, A.; Hébert, P.; Kohara, S.; Keen, D.; Hammouda, T.; Andrault, D. Topologically Ordered Amorphous Silica Obtained from the Collapsed Siliceous Zeolite, Silicalite-1-F: A Step toward "Perfect" Glasses. *J. Am. Chem. Soc.* **2009**, *13*, 12333–12338.

(2) Santoro, M.; Veremeienko, V.; Polisi, M.; Fantini, R.; Alabarse, F.; Arletti, R.; Quatieri, S.; Svitlyk, V.; van der Lee, A.; Rouquette, J. et al. Insertion and Confinement of H_2O in Hydrophobic Siliceous Zeolites at High Pressure. *The Journal of Physical Chemistry C* **2019**, *123*, 17432–17439.

(3) Mao, H.; Bell, P.; Shaner, J. t.; Steinberg, D. Specific volume measurements of Cu, Mo, Pd, and Ag and calibration of the ruby R1 fluorescence pressure gauge from 0.06 to 1 Mbar. *Journal of Applied Physics* **1978**, *49*, 3276–3283.

(4) Akahama, Y.; Kawamura, H. Pressure calibration of diamond anvil Raman gauge to 310 GPa. *Journal of Applied Physics* **2006**, *100*, 043516.

(5) Howie, R. T.; Gregoryanz, E.; Goncharov, A. F. Hydrogen (deuterium) vibron frequency as a pressure comparison gauge at multi-Mbar pressures. *Journal of Applied Physics* **2013**, *114*, 073505.

(6) Prescher, C.; Prakapenka, V. B. DIOPTAS: a program for reduction of two-dimensional X-ray diffraction data and data exploration. *High Pressure Research* **2015**, *35*, 223–230.

(7) Carvajal, J. R. Recent developments of the program FULLPROF, in commission on powder diffraction (IUCr). *Newsletter* **2001**, *26*, 12–19.

(8) Fei, Y.; Ricolleau, A.; Frank, M.; Mibe, K.; Shen, G.; Prakapenka, V. Toward an internally consistent pressure scale. *Proceedings of the National Academy of Sciences* **2007**, *104*, 9182–9186.

(9) Desbiens, N.; Boutin, A.; Demachy, I. Water condensation in hydrophobic silicalite-1 zeolite: a molecular simulation study. *The Journal of Physical Chemistry B* **2005**, *109*, 24071–24076.

(10) Coasne, B.; Galarneau, A.; Di Renzo, F.; Pellenq, R. Intrusion and retraction of fluids in nanopores: effect of morphological heterogeneity. *The Journal of Physical Chemistry C* **2009**, *113*, 1953–1962.

(11) Leachman, J. W.; Jacobsen, R. T.; Penoncello, S.; Lemmon, E. W. Fundamental equations of state for parahydrogen, normal hydrogen, and orthohydrogen. *Journal of Physical and Chemical Reference Data* **2009**, *38*, 721–748.

(12) McCarty, R. D.; Hord, J.; Roder, H. M. *Selected properties of hydrogen (engineering design data)*; US Department of Commerce, National Bureau of Standards, 1981; Vol. 168.

(13) Kunz, O.; Klimeck, R.; Wagner, W.; Jaeschke, M. *The GERG-2004 Wide-Range Reference Equation of State for Natural Gases and Other Mixtures GERG TM15 2007*; VDI-Verlag, 2007.

(14) Kowalczyk, P.; Terzyk, A. P.; Gauden, P. A.; Furmaniak, S.; Pantatosaki, E.; Papadopoulos, G. K. Intrinsic D_2/H_2 Selectivity of NaX Zeolite: Interplay between Adsorption and Kinetic Factors. *J. Phys. Chem. C* **2015**, *119*, 15373—-15380.

(15) Keskin, S. Adsorption, Diffusion, and Separation of CH_4/H_2 Mixtures in Covalent Organic Frameworks: Molecular Simulations and Theoretical Predictions. *J. Phys. Chem. C* **2012**, *116*, 1772—-1779.

(16) Liu, J.; Culp, J. T.; Natesakhawat, S.; Bockrath, B. C.; Zande, B.; Sankar, S. G.; Garberoglio, G.; Johnson, J. K. Adsorption, Diffusion, and Separation of CH_4/H_2 Mixtures in Covalent Organic Frameworks: Molecular Simulations and Theoretical Predictions. *J. Phys. Chem. C* **2007**, *111*, 9305—-9313.

(17) Radola, B.; Giraudet, M.; Bezverkhyy, I.; Simon, J. M.; Salazar, J. M.; Macaud, M.; Bellat, J. P. New force field for GCMC simulations of D_2/H_2 quantum sieving in pure silica zeolites. *Phys. Chem. Chem. Phys.* **2020**, *22*, 24561–24571.

(18) Dubbeldam, D.; Walton, K. S.; Vlugt, T. J. H.; Calero, S. Design, Parameterization, and Implementation of Atomic Force Fields for Adsorption in Nanoporous Materials. *Advanced Theory and Simulations* **2019**, *2*, 1900135.

(19) Darkrim, F.; Levesque, D. J. Monte Carlo simulations of hydrogen adsorption in single-walled carbon nanotubes. *The Journal of Chemical Physics* **1998**, *109*, 4981–4984.

(20) Desbiens, N.; Demachy, I.; Fuchs, A. H.; Kirsch-Rodeschini, H.; Soulard, M.; Patarin, J. Water condensation in hydrophobic nanopores. *Angewandte Chemie International Edition* **2005**, *44*, 5310–5313.

(21) Garberoglio, G.; Skoulidas, A. I.; Johnson, J. K. Adsorption of gases in metal organic materials: comparison of simulations and experiments. *The Journal of Physical Chemistry B* **2005**, *109*, 13094–13103.

(22) Olson, D.; Kokotailo, G.; Lawton, S.; Meier, W. Crystal structure and structure-related properties of ZSM-5. *The Journal of Physical Chemistry* **1981**, *85*, 2238–2243.

(23) Moshary, F.; Chen, N. H.; Silvera, I. F. Pressure dependence of the vibron in H_2, HD, and D_2: Implications for inter-and intramolecular forces. *Physical Review B* **1993**, *48*, 12613–12619.

(24) Loubeyre, P.; LeToullec, R.; Pinceaux, J. Raman measurements of the vibrational properties of H_2 as a guest molecule in dense helium, neon, argon, and deuterium systems up to 40 GPa. *Physical Review B* **1992**, *45*, 12844–12553.

(25) Hanfland, M.; Hemley, R.; Mao, H.; Williams, G. Synchrotron infrared spectroscopy

at megabar pressures: Vibrational dynamics of hydrogen to 180 GPa. *Physical Review Letters* **1992**, *69*, 1129–1132.

(26) Hanfland, M.; Hemley, R. J.; Mao, H.-k. Novel infrared vibron absorption in solid hydrogen at megabar pressures. *Physical Review Letters* **1993**, *70*, 3760–3763.
